# Microbiological and Sensorial Quality of Beef Meat (*Longissimus dorsi*) Marinated with Cinnamon Extract and Stored at Various Temperatures

**DOI:** 10.3390/foods11243971

**Published:** 2022-12-08

**Authors:** Che Jaafar Zawani, Mahmud Ab Rashid Nor-Khaizura, Nor Ainy Mahyudin, Mohammad Rashedi Ismail-Fitry, Nilesh Prakash Nirmal

**Affiliations:** 1 Department of Food Science, Faculty of Food Science and Technology, Universiti Putra Malaysia (UPM), Serdang 43400, Selangor, Malaysia; 2 Laboratory of Food Safety and Food Integrity, Institute of Tropical Agricultural and Food Security, Universiti Putra Malaysia (UPM), Serdang 43400, Selangor, Malaysia; 3 Department of Food Service and Management, Faculty of Food Science and Technology, Universiti Putra Malaysia (UPM), Serdang 43400, Selangor, Malaysia; 4 Department of Food Technology, Faculty of Food Science and Technology, Universiti Putra Malaysia (UPM), Serdang 43400, Selangor, Malaysia; 5 Institute of Nutrition, Mahidol University, 999 Phutthamonthon 4 Road, Salaya, Nakhon Pathom 73170, Thailand

**Keywords:** meat, *Pseudomonas* spp., storage temperature, cinnamon extract, antimicrobial, antioxidant, shelf-life extension

## Abstract

Meat spoilage caused by temperature abuse is a major problem for producers, retailers, and consumers that can generate large economic losses to industries. Microbial growth of *Pseudomonas* spp. is the main source of spoilage during storage. Cinnamon has antimicrobial properties that may potentially be used to reduce the spoilage caused by *Pseudomonas*. The objectives of this study were to determine the inhibitory effect of cinnamon extract (CE) against *Pseudomonas aeruginosa* (ATCC 27853) and evaluate the treatment of CE on meat quality during different storage temperatures (5 °C, 10 °C, 15 °C, and 25 °C). The anti-*Pseudomonas* result showed that 100% (*w*/*v*) CE concentration produced a 13.50 mm zone of inhibition in a disc diffusion assay. The minimum inhibitor concentration (MIC) of CE was noted at 25% (*v*/*v*), whereas the minimum bactericidal concentration (MBC) value was observed at 50% (*v*/*v*) concentration of CE. The time-kill showed the growth of *P. aeruginosa* decreased from 7.64 to 5.39 log CFU/mL at MIC concentration. Total phenolic content and IC_50_ value of the cinnamon extract was expressed as 6.72 ± 0.87 mg GAE/g extract and 0.15 mg/mL, respectively. When the meat was marinated with 50% (*v*/*v*) CE and stored at various temperatures, the total viable count (TVC) and growth of *Pseudomonas* spp. were lowered as compared to the control sample. However, the reduction in microbial count in all samples was influenced by the storage temperature, where the lowered microbial count was noted in the sample treated with CE and stored at 5 and 10 °C for 48 h. The pH of meat treated with or without CE ranged from pH 5.74 to 6.48. The sensory attributes of colour, texture, and overall acceptability have a significant difference, except for odour, between marinated meat and control. The results indicate that the use of cinnamon extract as the marination agent for meat could reduce the growth of *Pseudomonas* spp. and therefore assist in extending the shelf life of meat at 5 and 10 °C storage temperatures.

## 1. Introduction

Meat is a highly perishable food that can spoil in a relatively short time unless appropriate actions are taken (e.g., packaged, transported, and stored at refrigeration temperatures) [1]. Microbial growth and foul smell are the prime factors affecting the shelf-life and consequent consumer acceptance of fresh meat [2,3]. Factors affecting meat spoilage include intrinsic and extrinsic parameters, where the temperature is considered the most important factor. Hence, temperature abuse during any stage of the chill chain may result in an unexpected loss of quality and a significant decrease in meat shelf life [4,5].

Specific spoilage organisms (SSOs) or ephemeral spoilage organisms are the subjects of the theory of ‘succession’ of spoilage-related microbial groups [6]. According to Shao et al. [7], several studies have focused on meat with the purpose to describe the diversity of the spoilage-related microbial populations in response to dynamic storage conditions. The main flora liable for spoilage in meat products during aerobic storage are *Pseudomonads*, which are the Gram-negative psychotrophs that affect the quality and shelf life of the products and can be responsible for extensive economic losses [8,9]. According to Koutsomanis et al. [5] and Ercolini et al. [10], *Pseudomonas* was the specific spoilage organism of chilled meat due to its dominance, and they established a *Pseudomonas* growth model. 

In order to prevent the emergence of SSO in meat, various chemical food additives such as Butylated hydroxyanisole (BHA), butylated hydroxytoluene (BHT), and tertiary butyl hydroquinone (TBHQ) have been applied in the food industry and function to inhibit both microbial contamination and lipid oxidation [11]. These also reduce the occurrence of food poisoning and spoilage with antimicrobial agents [12]. There is a limitation allowance of the mixture of BHA and BHT for food application, which is less than 0.01% each by weight [13]. Besides, the concern of synthetic food additives discovered was that the use of these compounds led to health risks [14,15]. Hence, various studies have been conducted to find natural antioxidant and antimicrobial agents to replace BHA and BHT in food products [16,17].

*Cinnamomum* is a genus in the family Lauraceae, which is generally a spice in cooking. One of the major species of *Cinnamomum* is *Cinnamomum zeylanicum* Nees (cinnamon) and is also known as *Cinnamomum verum* J.S. Presl. This spice has been widely used in many applications in the perfumery, food, and pharmaceutical industries [18]. People commonly know them by their commercial name, cinnamon stick. Cinnamon and cassia are rich in essential oils, mainly cinnamaldehyde [19,20] and eugenol, which can inhibit microbial growth [19]. According to Lu et al. [21], barks of *Cinnamomum* plants contained few components as condensed tannins, which are dimeric, trimeric, and higher oligomeric, polymeric proanthocyanidins (flavan-3-ols). In vitro studies have recently shown that *C. verum* essential oil effectively inhibits food spoilage and the growth of pathogenic bacteria [20,22]. Despite its antimicrobial activity, this oil has several positive impacts on health [19,23]. Thus, it is deemed an effective alternate natural food preservative agent. In this study, the sample used was fresh meat from the *Longissimus dorsi* (striploin), which is one of the important parts of beef cattle. Therefore, the shelf life extension of this part of meat using natural plant extract is worth exploring. This study aimed to evaluate the antimicrobial properties of the cinnamon extract against *Pseudomonas aeruginosa* ATCC 27853. Then, it aimed to determine the effect of cinnamon extract treatment on meat quality during various storage temperatures (5, 10, 15, and 25 °C). The output of this work could be essential to the meat industry as well as a guide for future research.

## 2. Materials and Methods

### 2.1. Cinnamon Extract Preparation (CE)

The cinnamon powder was bought from Suri Niaga Resources, Kuala Lumpur and stored at room temperature before being used. A total of 5 g of cinnamon powder was soaked in 95 mL sterile distilled water for 1 h at room temperature with occasional stirring followed by gentle boiling for 2 min on a plate heater equipped with a magnetic stirrer. The extract was obtained by cooling and filtration through Whatman No. 4 filter paper and kept in the dark before being used. The filtrate was regarded as 100% (*w*/*v*) concentration of extract and was further diluted to obtain different concentrations (*v*/*v*) by mixing with appropriate volumes of sterile distilled water [24].

### 2.2. Anti-Pseudomonas Activity of the CE

#### 2.2.1. Preparation of Bacterial Strains

The bacterial strain that causes spoilage in meat was used as a test strain: *Pseudomonas aeruginosa* American Type Culture Collection 27,853 was obtained from the ATCC (Manassas, VA, USA) and subcultured weekly on trypticase soy agar (TSA) (Pronadisa, Madrid, Spain). Strains were preserved frozen in cryovials at −80 °C. Trypticase soy broth (Difco-0369-01-4) was used for the activation of bacterial cultures, and Mueller Hinton agar (OXOID CM 337) for antimicrobial activity trials was used.

#### 2.2.2. Disc Diffusion Assay

The CE was tested for antibacterial activity using the disc diffusion method as described by the Clinical and Laboratory Standard Institute [25]. Diameter discs of 6 mm were impregnated with 10 µL of different concentrations (20, 40, 60, 80 and 100% (*w*/*v*)) of CE before being placed on the inoculated agar plates. *Pseudomonas aeruginosa* ATCC 27,853 was streaked on Mueller Hinton agar plates (MHA, Difco, Sparks, MD, USA) using a sterile cotton swab. When the inoculum dried, the impregnated discs were placed on the agar using forceps dipped in ethanol and flamed, gently pressed down to ensure contact. Gentamycin (a reference antibiotic) was used as a positive control. The plates were incubated at 37 °C overnight for 24–48 h. Evidence of a clear zone indicates bacterial growth inhibition, and the diameter was measured in mm. The antibacterial activity for disc diffusion assay was performed in triplicate. 

#### 2.2.3. Minimal Inhibitory and Minimal Bactericidal Concentration (MIC and MBC)

MIC was determined using the broth microdilution assay [26]. A total of 100 µL of scalar dilution was transferred from the suspension of a 100 µL extract with 100 µL of Mueller–Hinton broth and inoculum from the 12th well to the 3rd well with the 2nd well as positive control and the 1st well as a negative control because no cinnamon extract was added. After 24 h incubation time at 37 °C, the 96-well plates were visually observed. Bacterial growth was detected by button formation on the wall of the well plate. 

From the MIC well, media from each well showing no visible growth was removed and subcultured onto Mueller–Hinton agar plates. The plates were incubated at 30 °C for 24 h until growth was seen in the growth control plates to determine the MBC.

#### 2.2.4. Time-Kill Analysis

Time-kill assay was performed to determine the time taken for the microorganisms to be killed by the cinnamon extract, and it was performed following the method of Rukayadi et al. [27]. Mueller–Hinton broth was used, and bacterial inoculum was adjusted between 6 to 8 log CFU/mL. The final concentrations of cinnamon were 0 MIC, 0.5 × MIC, and 1 × MIC aliquots. The cultures were then incubated at 37 °C with an agitation of 200 rpm. At predetermined intervals of 0, 0.5, 1, 2, 4, and 6 h, 1 mL aliquots were serially diluted in 1% (*w*/*v*) phosphate-buffered saline (PBS) and plated onto Mueller–Hinton (Oxoid, UK) agar plates. The plates were incubated at 37 °C for 24 h, and the number of colonies was counted. The assays were carried out in triplicate. The graph of log_10_ CFU/mL was plotted against time.

#### 2.2.5. Total Phenolic Content (TPC)

A total of 0.5 mL of cinnamon extract was added to 0.5 mL of Folin–Ciocalteu reagent and was transferred to a 96-well microplate. The mixture was mixed well and stayed in the dark for 5 min. Then, 10 mL of 7% sodium carbonate was added to the mixture. The mixture was then allowed to react at room temperature. Then, the microplate was placed in a microplate reader. The absorbance was measured at 765 nm using a UV-1650 PC UV-Vis spectrophotometer (Shimadzu, Kyoto, Japan). The TPC was calculated from a calibration curve of gallic acid and expressed as milligrams of gallic acid equivalent (GAE) per gram of cinnamon extract. 

#### 2.2.6. DPPH Radical Scavenging Method

The antioxidant activity of the cinnamon extract was measured based on its scavenging activities towards the stable 1,1-diphenyl-2-picrylhydrazyl (DPPH) radical. The extract was dissolved in methanol and 0.5 mL of extract at different concentrations was added to 3.5 mL DPPH solution (25 µg/mL). The mixture was left to stand at room temperature for 30 min. The absorbance was measured at 517 nm using a UV-1650 PC UV-Vis spectrophotometer (Shimadzu, Kyoto, Japan). Percent free radical scavenging capacity (%I) was calculated as a percentage of DPPH inhibition using the following equation:

% I = ((A_blank_ − A_extract_)/A_blank_) × 100

where A_blank_ is the absorbance of the DPPH solution with methanol and A_extract_ is the absorbance of the solution with extract expressed as IC_50_, the concentration of extract required to inhibit fifty per cent of DPPH. The IC_50_ value was calculated from the log concentration of extract against the percentage of inhibition.

### 2.3. CE treatment on Meat during Different Storage Temperatures 

#### 2.3.1. Meat Treatment

Fresh beef meat (*Longissimus dorsi*) that was less than 12 h after slaughter was bought from Pasar Borong Seri Kembangan and was used for this study. The meat was transported to the laboratory within 1 h of being purchased and held at 4 °C for 1 to 2 h. Each batch was further cut into portions of 100 g of meat with approximately 1 cm of thickness and placed inside a zip lock bag with 50% (*v*/*v*) cinnamon extract. The marinade was properly homogenised to all sides of the meat and the package was sealed and stored. A control sample without any marinade was prepared and stored at the same storage temperature as the marinated samples. Packaged meat was stored under controlled isothermal conditions (5, 10, 15, and 25 °C) in high-precision (±0.2 °C), low-temperature incubators (model MIR 153; Sanyo Electric Co., Ora-Gun, Gunma Japan). Microbiological analyses were carried out for the marinated and non-marinated meat stored at 5 °C, 10 °C, 15 °C, and 25 °C for 48 h. Analyses were carried out every 6 h for the first 24 h and every 12 h for the consequent 48 h intervals. The temperature variation including 25 °C was carried out to imitate the condition of temperature abuse that may occur during meat storage. Therefore, the impact of temperature abuse on microbial growth could be revealed. The sensory evaluation was carried out for marinated and non-marinated meat stored at 5 °C for 24 h. 

#### 2.3.2. Microbiological Analysis 

A total of 10 g of marinated and non-marinated meat sample was homogenized in a stomacher (Stomacher Lab-Blender 400, West Sussex, UK) with 90 mL of 0.1% (*w*/*v*) peptone water. A serial dilution was prepared. Up to a dilution factor of 10^−5^ × 0.1 mL of each serial dilution was pipetted onto the Plate Count Agar (OXOID, Hampshire, UK) for total viable count (TVC) and *Pseudomonas* base agar (OXOID, Hampshire, UK) with *Pseudomonas* C-F-C supplement SR0103E for the *Pseudomonas* count. The inoculum was spread uniformly by using a sterile spreader. The plates were incubated at 35 °C for 48 h. All plates were inspected visually for colony type and morphological characteristics associated with the growth medium. Microbial colonies were counted and expressed as log CFU (colony forming units)/g of meat. 

#### 2.3.3. pH Determination

A total of 10 g of marinated and non-marinated meat sample was homogenized in 100 mL distilled water. The pH values were measured at 28 ± 2 °C using a digital pH meter (Mettler Toledo, Columbus, OH, USA) calibrated at pH 4.0, 7.0, and 9.0 using standard buffer solutions.

#### 2.3.4. Sensory Evaluation

Sensory evaluation was conducted using 30 panellists composed of students and lecturers of Universiti Putra Malaysia. The ratio of panellists was 70% female and 30% male, aged between 21 to 48 years old, and had basic knowledge of meat quality. Informed consent was obtained from all panellists involved in the study. Panellists were asked to score the sensory properties of the marinated and non-marinated meat based on visual inspection using four attributes; odour, colour, texture, and overall acceptance [28]. The intensity for each attribute was rated on a 9-point hedonic scale. Sensory consumer analysis was undertaken in the panel booths at the food sensory evaluation laboratory that conforms to ISO 8589 [29] international standards. 

### 2.4. Statistical Analysis

Experiments were performed in triplicate using different batches of meat and the specific analyses were conducted with at least two repetitions. Results were expressed as mean with standard deviations. The significant differences in treatment were obtained by one-way analysis of variance (ANOVA) using Tukey’s test.

## 3. Results and Discussion

### 3.1. Anti-Pseudomonas Activity 

The antibacterial activity is important to determine the ability of plant extract to reduce and inhibit the spoilage bacteria, in this case focusing on *Pseudomonas* inhibition. The anti-*Pseudomonas* activity of the cinnamon extract was measured using the disc diffusion agar. Increasing the concentration of cinnamon extract showed an increasing inhibition of *Pseudomonas aeruginosa* (Table 1). It has been proposed that a diameter in the range of more than 16 mm is highly active, 12 to 16 mm is moderately active, and less than 12 mm has no antimicrobial activity [30,31]. The cinnamon extract at 100% (*w*/*v*) concentration showed a 13.500 ± 0.837 inhibition zone and could be determined moderately active against *Pseudomonas aeruginosa*. However, the minimum inhibitory concentration (MIC) was observed at 25% (*v*/*v*) of CE, whereas the minimum bactericidal concentration (MBC) was achieved at 50% (*v*/*v*). MBC value was determined to be the lowest concentration of the agar plate at which bacterial growth was absent and no bacteria grew after inoculation in the broth media. The chemical constituents in hot water extraction for cinnamon are reported as carbohydrates, steroids, alkaloids, and saponin [32]. These phytochemicals are important because they are responsible for antibacterial activities. Phytochemicals are certified as GRAS (Generally Recognised as Safe) [14], and pleasantly accepted by the majority of consumers to be added to food, in comparison with synthetic preservatives. 

**Figure 1:**
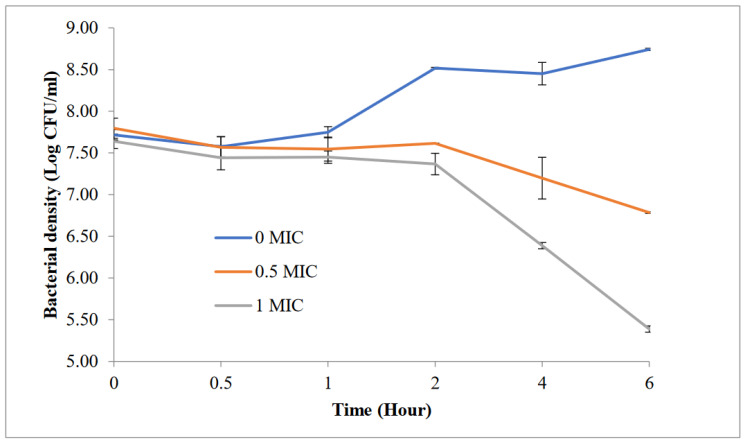
Time-kill of *Pseudomonas aeruginosa* ATCC 27,853 in the presence of cinnamon extract. CE concentrations: 0% (*v*/*v*); 0.5% (*v*/*v*); 1.0% (*v*/*v*). Bars show the SD of two independent experiments and a total of 4 replicates (*n* = 4).

A time-kill assay was performed to evaluate the inhibition potential of the cinnamon extract against *P. aeruginosa* (Figure 1). At the half MIC value of the cinnamon extract, an almost 1-log reduction of *P. aeruginosa* was observed after 6 h. At concentrations of the MIC, a 2.25-log reduction was achieved after 6 h, in which the initial count of *P. aeruginosa* was 7.64 log CFU/mL reduced to 5.39 log CFU/mL. From the time-kill assay in the present study, the cinnamon extract exhibited bacteriostatic effects within 6 h of incubation. This result is in line with Hacioglu et al. [33], who also found that cinnamon extract showed a bacteriostatic effect rather than bactericidal effect towards reducing *P. aeruginosa* growth. Gilani and Najafour [34] reported that bioactive compounds of the cinnamon extract using water extraction that might possess antibacterial effects are cinnamaldehyde and cinnamic acid. 

### 3.2. Total Phenolic Content and Antioxidant Activity

Total phenolic content is an analysis to measure the amount of phenolic content in one sample. In order to calculate the TPC of cinnamon extract, a gallic acid standard graph with standard curve y = 0.0466x + 0.3233, where R^2^ = 0.9924, was developed. Based on the standard graph, the total phenolic content of the cinnamon extract was expressed as 6.72 ± 0.87 mg GAE/g extract. This was slightly higher compared with the study by Sana et al. [35], in which they obtained 5.48 mg GAE/g for cinnamon extract. The cinnamon extract was expected to have antioxidant and antibacterial activities. This could relate to the ability of the extract to inhibit the growth of *Pseudomonas* in meat when being applied as a marinade. 

The scavenging of radicals was found to be high, and the 50% inhibitory concentration (IC_50_ value) was 0.15 mg/mL cinnamon extract. The IC_50_ value of the standard ascorbic acid was 0.03 mg/mL. Normal classification of a compound antioxidant activity generally follows the following criteria: IC_50_ < 0.05 mg/mL is considered a very powerful antioxidant; IC_50_ 0.05–0.10 mg/mL as a strong antioxidant; intermediate antioxidants (0.10–0.15 mg/mL); and weak antioxidants (IC_50_ 0.15–0.20 mg/mL). Based on these criteria, the results obtained in this study were classified as very intermediate antioxidants. Commonly, the total phenolic compound is correlated to antioxidant activity. This is because they have the capability to destroy free radicals by transferring their electrons to react with the free radicals [36]. The antioxidant properties of cinnamon extract might be attributed to the higher phenolic compounds present. The total phenolic compounds and antioxidant properties in the cinnamon extract can be an essential parameter response to the presence of antimicrobial activities.

### 3.3. Effect of CE Treatment on Meat during Different Storage Temperatures

#### 3.3.1. Microbiological Changes

The microbiological changes are crucial to reveal the effectiveness of marination in reducing the total viable count and *Pseudomonas* spp. During meat storage. The changes in total viable count (TVC) and *Pseudomonas* count of raw meat treated with or without cinnamon extracts (50% (*v*/*v*)) during various storage temperatures are presented in Table 2 and Table 3, respectively. Table 2 shows the effect of cinnamon extracts on the total viable count, TVC (log CFU/g) of cinnamon-marinated meat, and control (non-marinated meat) samples stored at various temperatures. The initial TVC value of marinated and non-marinated meat was found to be 5.31 and 5.84 log CFU/g of the sample, respectively. In general, the TVC value of all samples increases with increasing storage time and temperatures. However, the increase in TVC value was lowered in the sample treated with CE and stored at 5, 10, and 15 °C temperatures. The control sample showed a higher TVC value than the 8 log CFU/g sample during 10, 15, and 25 °C storage temperatures at 48, 18, and 12 h of storage time, respectively. At the end of the storage day, there was no significant change in the TVC value of all samples stored at 25 °C. At room temperature (25 °C), CE was able to reduce TVC value up to 7.90 log CFU/g for 12 h only. CE was best able to protect the meat from the microbial count at 5 and 10 °C even after 48 h compared to the control (*p* <0.05). The sample treated with CE and stored at 15 °C was reported with less than 8 log CFU/g for 36 h of storage, whereas the control sample sustained that level for 12 h only (*p* < 0.05). The results indicated that storage temperature influences the microbial count of the meat during increased storage time, whereas treatment of the meat with CE retards the increasing total viable count of the meat during different storage temperatures compared to the control. 

Spices such as cinnamon have antibacterial properties, which can have a positive impact on meat [37]. Cinnamon inhibits microbes in a variety of ways, including by rupturing the cell wall through the action of antioxidant compounds, disrupting the cytoplasmic membrane, disrupting cellular components through leakage, changing fatty acid and phospholipid constituents, affecting DNA and RNA formation, and preventing protein translocation [38]. Cinnamon oil was found to be more effective than other ingredients in reducing microbial counts, according to Guiterrez et al. [39]. This supports our results, in which lowered TVC values were reported on meat treated with CE.

Table 3 represents the *Pseudomonas* spp. count (log CFU g) in meat samples treated with or without CE during storage at 5, 10, 15, and 25 °C for 48 h The initial count of *Pseudomonas* spp. was noted to be 4.26 and 4.96 log CFU g samples for CE-treated meat and control, respectively. This indicated that treatment of the meat with CE immediately reduces the initial *Pseudomonas* spp. load on the meat. Similar to the TVC, the growth of *Pseudomonas* spp. was influenced by storage temperature. At 5 °C storage temperature, the *Pseudomonas* spp. count was lowered by less than 6 log CFU/g for both samples (CE treated and control). However, CE provides higher retardation to *Pseudomonas* spp. compared to the control for 36 h (*p* < 0.05). When samples were stored at 10 °C, log 6 count was noted after 6 h in the control, whereas the CE-treated meat sample showed the same growth after 48 h. At higher temperatures of 15 and 25 °C, the control sample attained a log 8 count after 24 h of storage, while the CE-treated sample stored at 25 °C showed a log 8 count after 36 h, whereas CE-treated samples stored at 15 °C did not attain a log 8 count even after 48 h. These results clearly suggested that CE was able to reduce the *Pseudomonas* spp. growth in meat samples immediately after treatment as well as during storage at various temperatures. *Pseudomonas* spp. is known to produce hydrogen sulphide and other spoilage compounds, which is the indicator of food spoilage [40]. 

The results show that the *Pseudomonas* values decreased significantly (*p* < 0.05) with the addition of cinnamon extract compared to non-marinated meat. No limitation has been stated for *Pseudomonas* spp., as this is spoilage bacteria rather than pathogenic bacteria. However, the *Pseudomonas* count should be at the lower count, which is suggested below 10^3^ [41,42] in order to delay meat spoilage during storage. *Pseudomonas* count is directly associated with the period of storing meat in the refrigerator, and meat spoilage occurs when the number of *Pseudomonas* ranges from 10^7^ to 10^8^ [41].

Fresh meat represents an optimum medium for microbial growth because of its unique biochemical properties and nutrient composition. Deterioration happens progressively from slaughter until consumption. The shortened shelf life is due to microbial growth and/or rancidity development, which is strongly influenced by the initial beef quality, package parameters, and storage conditions [43]. Microbial growth is the most important factor in the spoilage of fresh meat, and this is followed by colour deterioration. Different types of spoilage microorganisms may be introduced into and on the surface of fresh meat during slaughtering and processing, which causes rapid spoilage, great loss of valuable protein, and also affects human health [44]. 

An in vitro study has shown that *C. verum* essential oil effectively inhibits food spoilage and the growth of pathogenic bacteria [22]. Essential oils are a complex mixture of secondary metabolites produced by aromatic plants. These essential oils and their components also show antimicrobial properties and could thus be useful for the conservation of foodstuffs [45]. *C. verum* essential oil can inhibit the *Pseudomonas* spp., particularly *Pseudomonas aeruginosa*, by damaging the cellular membrane of *P. aeruginosa*, which leads to cell death. Exposure to this oil-induced alteration in the bacterial membrane of *P. aeruginosa* led to the collapse of membrane potential, as demonstrated by bis-oxonol staining and loss of membrane-selective permeability, as indicated by the efflux of K^+^ and propidium iodide accumulation. Thus, respiratory activity was inhibited, leading to cell death [46]. The essential oil extracted from *C. verum* bark is composed mainly of cinnamaldehyde (79.3% *w*/*w*) [20] and eugenol (11.9% *w*/*w*).

#### 3.3.2. pH

Monitoring the pH of meat during storage could reflect on the safety and quality of the food. Table 4 shows the pH of cinnamon-marinated meat stored at temperatures of 5, 10, 15, and 25 °C. The initial value of cinnamon-marinated meat was found to be pH 6.10. The pH values of the marinated meat samples stored at various temperatures were found to decrease from pH 6.10 to 5.98 at 5 °C for 6 h and from pH 6.10 to 5.74 at 25 °C for 18 h, and increase from pH 6.10 to 6.19 at 10 °C for 12 h and from pH 6.10 to 6.20 at 15 °C. The pH increase (*p* < 0.05) during all storage temperatures may be due to the utilization of the amino acids by bacteria, which are released during protein degradation because the stored glucose has been depleted. Accumulation of ammonia and the products of amino acid decomposition increase pH [47]. Cinnamaldehyde is the most important chemical component of cinnamon, and it has been shown to inhibit meat spoilage bacteria [48]. Also, the content of cinnamaldehyde found in cinnamon causes the acidity of the marinated meat to decrease the pH. This is evident in the results where concomitant increase and decrease in pH was observed during all storage temperatures.

#### 3.3.3. Sensory Analyses

Sensory analyses for any changes in food formulation are essential to attain consumer acceptability. The sensory evaluation results presented in Table 5 show the score between cinnamon-marinated and control (non-marinated meat) samples for odour, colour, texture, and overall acceptance after 24 h of storage at 5 °C. The comparison between the marinated samples and the control in terms of colour showed that the marinated meat has a slightly brown colour, and the control has a red colour. In terms of odour, the marinated meat has a smell of cinnamon, compared to the control, which has fresh meat odours, and for texture, marinated was less firm than the control. The sensory score for the odour showed no significant difference (*p* < 0.05) between marinated and non-marinated meat. However, the non-marinated meat scored higher (*p* < 0.05) than the cinnamon-marinated meat for colour, texture, and overall acceptance. The colour of cinnamon-marinated meat turns slightly brownish due to the cinnamon extract’s natural colour. This could be the reason for the slightly lower score for the cinnamon-marinated meat. These results align with Van Haute et al. [49] and Putra et al. [50], whose sensory score for marinated meat is lower than that of non-marinated. The samples that were treated with the essential oil including cinnamon marinade had a significantly lower hedonic value than those without essential oil [49]. However, the sensory evaluation of marinated meat normally improves when it undergoes cooking processes such as grilling, baking, or frying. The cinnamon marinade is expected to improve the odour, colour, and texture of the cooked meat due to its pungent and aromatic plant substances [51]. Generally, when the plant extracts are applied in food formulations, the sensorial impact of these extracts is a limitation on the quantity of extract that can be applied. The alternatives to lessen this impact are the usage of an appropriate amount of extract, the use of bioactive compounds instead of the extract [49], and nanoemulsion formulation, which could improve the sensory evaluation of food treated with the plant extract.

## 4. Conclusions

Cinnamon extract was proved to inhibit the presence of *Pseudomonas aeruginosa* at 50% (*v*/*v*) concentration. The antibacterial activity of CE was related to the presence of TPC and antioxidant activity. CE-marinated meat showed slower growth for both total viable count and *Pseudomonas* spp. than control, at 5 °C, followed by CE-marinated meat storage at 10 and 15 °C, whereas no reduction occurred at 25 °C. CE-marinated meat has a better shelf life when stored at a lower temperature (5 and 10 °C) with not many changes in pH values; however, the colour of meat is a bit affected due to the extract’s natural colour. Overall results suggest that CE inhibited the growth of *P. aeruginosa* and CE treatment to meat significantly reduced the TVC as well as *Pseudomonas spp.* during storage at various temperatures. However, the colour and overall acceptance of meat were negatively affected by the addition of CE compared to the control. Hence, a nanoemulsion formulation of CE was suggested to reduce the negative effect of CE colour and improve the overall acceptance of the food product. 

## Figures and Tables

**Table 1:** Antibacterial activity of cinnamon extract for *Pseudomonas aeruginosa* ATCC 27853.

Cinnamon Extract Concentration (%)	Zone of Inhibition (mm)
**20**	7.75 ± 0.42 ^a^
**40**	8.83 ± 0.42 ^b^
**60**	9.67 ± 0.82 ^bc^
**80**	10.50 ± 0.84 ^cd^
**100**	13.50 ± 0.84 ^d^
**Control (Gentamicin)**	15.00 ± 0.32 ^e^

Different small letters represent significant differences (*p* < 0.05) between the concentration of cinnamon extract.

**Table 2:** Total viable count, TVC (log CFU/g) of cinnamon-marinated meat and control (non-marinated meat) stored at 5, 10, 15, and 25 °C.

Time (h)	5 °C		10 °C		15 °C		25 °C	
	Marinated	Control	Marinated	Control	Marinated	Control	Marinated	Control
**0**	5.31 ± 0.51 Aa	5.84 ± 0.06 Ba	5.31 ± 0.51 Aa	5.84 ± 0.06 Ba	5.31 ± 0.51 Aa	5.84 ± 0.06 Ba	5.31 ± 0.51 Aa	5.84 ± 0.06 Ba
**6**	6.52 ± 1.28 Ab	5.80 ± 0.08 Ba	5.47 ± 0.70 Aa	6.81 ± 0.04 Bb	5.65 ± 0.77 Aab	7.78 ± 0.00 Bb	6.68 ± 0.30 Ab	7.88 ± 0.27 Bb
**12**	6.60 ± 1.00 Ab	6.54 ± 0.06 Ab	6.26 ± 0.27 Abc	7.37 ± 0.06 Bc	5.99 ± 0.65 Ab	7.85 ± 0.03 Bb	7.90 ± 0.17 Ac	8.08 ± 0.12 Bbc
**18**	6.29 ± 0.89 Ab	6.28 ± 0.09 Ac	6.09 ± 0.97 Ab	7.34 ± 0.07 Bcd	6.96 ± 0.27 Ac	8.06 ± 0.06 Bbc	8.36 ± 0.07 Ad	8.58 ± 0.35 Ac
**24**	6.74 ± 0.56 Abc	7.22 ± 0.13 Bd	6.22 ± 0.75 Abc	7.52 ± 0.11 Bcd	7.35 ± 0.22 Acd	8.97 ± 0.04 Bc	8.43 ± 0.30 Ad	8.84 ± 0.03 Ad
**36**	6.83 ± 0.61 Ac	7.57 ± 0.01 Bde	6.62 ± 0.79 Ac	7.73 ± 0.15 Bd	7.71 ± 0.28 Ad	8.50 ± 0.10 Bbc	8.62 ± 0.25 Ad	8.87 ± 0.03 Ad
**48**	6.61 ± 0.54 Ac	7.64 ± 0.11 Be	6.60 ± 0.64 Ac	8.18 ± 0.01 Be	8.23 ± 0.19 Ae	8.94 ± 0.09 Bc	9.17 ± 0.17 Ae	9.19 ± 0.04 Ae

Different capital letters represent significant differences (*p* < 0.05) between the marinated meat and the control (with a similar temperature of storage). Different small letters represent significant differences (*p* < 0.05) between the times of storage.

**Table 3:** *Pseudomonas* spp. count (log CFU/g) of cinnamon-marinated meat and control (non-marinated meat) stored at 5, 10, 15, and 25 °C.

Time (h)	5 °C		10 °C		15 °C		25 °C	
	Marinated	Control	Marinated	Control	Marinated	Control	Marinated	Control
**0**	4.24 ± 0.69 Aa	4.96 ± 0.60 Aa	4.24 ± 0.69 Aa	4.96 ± 0.60 Ba	4.24 ± 0.69 Aa	4.96 ± 0.60 Aa	4.24 ± 0.69 Aa	4.96 ± 0.60 Aa
**6**	3.79 ± 0.62 Aa	5.12 ± 0.01 Ba	4.09 ± 0.91 Aa	5.97 ± 0.02 Bb	4.13 ± 0.84 Aa	4.65 ± 0.12 Aa	4.78 ± 0.11 Aa	5.50 ± 0.15 Ba
**12**	4.38 ± 0.28 Aab	5.07 ± 0.01 Ba	4.82 ± 0.17 Aab	6.92 ± 0.04 Bc	4.66 ± 0.94 Aab	6.73 ± 0.10 Bb	5.94 ± 0.16 Ab	6.52 ± 0.12 Bb
**18**	4.99 ± 0.61 Abc	5.30 ± 0.00 Aab	4.41 ± 1.02 Aab	7.01 ± 0.05 Bc	5.30 ± 0.13 Ab	6.49 ± 0.10 Bb	7.21 ± 0.43 Ac	6.90 ± 0.35 Ab
**24**	5.37 ± 0.18 Ac	5.28 ± 0.03 Ab	4.87 ± 0.57 Aab	7.20 ± 0.04 Bcd	6.18 ± 0.22 Ac	8.15 ± 0.05 Bcd	7.85 ± 0.43 Acd	7.92 ± 0.03 Ac
**36**	4.89 ± 0.18 Abc	5.79 ± 0.01 Bc	4.14 ± 1.85 Aab	7.21 ± 0.18 Bd	6.66 ± 0.09 Ac	8.03 ± 0.03 Bc	8.35 ± 0.48 Ad	8.11 ± 0.03 Acd
**48**	5.39 ± 0.22 Ac	5.86 ± 0.02 Bc	5.94 ± 0.42 Ac	7.36 ± 0.00 Bd	7.46 ± 0.32 Ad	8.12 ± 0.10 bd	8.80 ± 0.25 Ad	8.20 ± 0.04 Bd

Different capital letters represent significant differences (*p* < 0.05) between the marinated and the control (within a similar temperature of storage). Different small letters represent significant differences (*p* < 0.05) between the time of storage.

**Table 4:** pH of cinnamon-marinated meat stored at 5, 10, 15, and 25 °C.

Storage Time (h)	5 °C	10 °C	15 °C	25 °C
**0**	6.10 ± 0.09 ^a^	6.10 ± 0.09 ^a^	6.10 ± 0.09 ^a^	6.10 ± 0.09 ^a^
**6**	5.98 ± 0.10 ^ab^	6.15 ± 0.21 ^a^	6.32 ± 0.40 ^ab^	6.06 ± 0.30 ^a^
**12**	6.10 ± 0.18 ^a^	6.19 ± 0.30 ^a^	6.20 ± 0.26 ^a^	5.93 ± 0.32 ^a^
**18**	6.05 ± 0.19 ^a^	5.95 ± 0.14 ^ab^	5.90 ± 0.13 ^a^	5.74 ± 0.53 ^a^
**24**	5.90 ± 0.07 ^ab^	6.06 ± 0.12 ^ab^	6.05 ± 0.15 ^a^	5.87 ± 0.61 ^a^
**36**	5.86 ± 0.08 ^b^	6.03 ± 0.12 ^ab^	5.96 ± 0.26 ^a^	6.02 ± 0.50 ^ab^
**48**	5.90 ± 0.12 ^ab^	6.11 ± 0.23 ^a^	6.05 ± 0.32 ^a^	6.48 ± 0.39 ^b^

Different small letters represent significant differences (*p* < 0.05) between the time of storage.

**Table 5:** Sensory of cinnamon-marinated meat and control (non-marinated meat) after 24 h.

Sample	Odour	Colour	Texture	Overall Acceptance
**Cinnamon-marinated meat**	4.617 ± 1.460 ^a^	4.233 ± 0.989 ^b^	4.183 ± 1.441 ^b^	4.450 ± 1.177 ^b^
**Non-marinated meat**	4.883 ± 1.127 ^a^	5.550 ± 1.117 ^a^	4.967 ± 1.224 ^a^	5.183 ± 0.966 ^a^
***p*** **-value**	0.432	0.000	0.027	0.012

Different small letters represent significant differences (*p* < 0.05) between the marinated and non-marinated (control) meat within the same sensory parameter.

## Data Availability

The data will be available from the corresponding author on request.

## References

[1] Bogataj D., Hudoklin D., Bogataj M., Dimovski V., Colnar S. (2020). Risk mitigation in a meat supply chain with options of redirection. Sustainability.

[2] Jiang J., Xiong Y.L. (2016). Natural antioxidants as food and feed additives to promote health benefits and quality of meat products: A review. Meat Sci..

[3] Jakobsen M., Bertelsen G. (2000). Colour stability and lipid oxidation of fresh beef. Development of a response surface model for predicting the effects of temperature, storage time, and modified atmosphere composition. Meat Sci..

[4] Ndraha N., Hsiao H.I., Vlajic J., Yang M.F., Lin H.T.V. (2018). Time-temperature abuse in the food cold chain: Review of issues, challenges, and recommendations. Food Control.

[5] Koutsoumanis K., Stamatiou A., Skandamis P., Nychas G.-J.E. (2006). Development of a microbial model for the combined effect of temperature and pH on spoilage of ground meat and validation of the model under dynamic temperature conditions. Appl. Environ. Microbiol..

[6] Pennacchia C., Ercolini D., Villani F. (2011). Spoilage-related microbiota associated with chilled beef stored in air or vakuum pack. Food Microb..

[7] Shao L., Chen S., Wang H., Zhang J., Xu X., Wang H. (2021). Advances in understanding the predominance, phenotypes, and mechanisms of bacteria related to meat spoilage. Trends Food Sci. Technol..

[8] Wang G., Tang W., Ma F., Wang H., Xu X., Qiu W. (2021). AprD is important for extracellular proteolytic activity, physicochemical properties and spoilage potential in meat-borne *Pseudomonas fragi*. Food Control.

[9] Widders P.R., Coates K.J., Warner S., Beattie J.C., Morgan I.R., Hickey M.W. (1995). Controlling microbial contamination on beef and lamb meat during processing. Aust. Vet. J..

[10] Ercolini D., Russo F., Nasi A., Ferranti P., Villani F. (2009). Mesophilic and psychrotrophic bacteria from meat and their spoilage potential in vitro and in beef. Appl. Environ. Microbiol..

[11] Oswell N.J., Thippareddi H., Pegg R.B. (2018). Practical use of natural antioxidants in meat products in the US: A review. Meat Sci..

[12] Su M.S., Shyu Y.T., Chien P.J. (2008). Antioxidant activities of citrus herbal product extracts. Food Chem..

[13] Food and Agriculture Organization (FAO) Current Market Situation and Medium-Term Outlook. Paper Presented at the Seventeenth Session of the Intergovernmental Group on Tea, 29 November–1 December 2006. http://www.fao.org.

[14] Lucera A., Costa C., Conte A., Del Nobile M.A. (2012). Food applications of natural antimicrobial compounds. Front. Microbiol..

[15] Das A.K., Anjaneyulu A.S.R., Biswas S. (2006). Effect of carnosine preblending on the quality of ground buffalo meat. Food Chem..

[16] Batiha G.E.S., Hussein D.E., Algammal A.M., George T.T., Jeandet P., Al-Snafi A.E., Tiwari A., Pagnossa J.P., Lima C.M., Thorat N.D. (2021). Application of natural antimicrobials in food preservation: Recent views. Food Control.

[17] Aksu M.I., Kaya M. (2005). The effect of α-tocopherol and butylated hydroxyanisole on the colour properties and lipid oxidation of kavurma, a cooked meat product. Meat Sci..

[18] Lee R., Balick M.J. (2005). Sweet wood–cinnamon and its importance as a spice and medicine. Explore.

[19] Boughendjioua H., Djeddi S. (2018). Study of The Organoleptic and Physicochemical Properties of Cinnamon Essential Oil (*Cinnamomum zeylanicum*). Am. J. Life Sci. Res..

[20] Singh G., Maurya S., deLampasona M.P., Catalan C.A.N. (2007). A comparison of chemical, antioxidant and antimicrobial studies of cinnamon leaf and bark volatile oils, oleoresins and their constituents. Food Chem. Toxicol..

[21] Lu Z., Jia Q., Wang R., Wu X., Wu Y., Huang C., Li Y. (2011). Hypoglycemic activities of A-and B-type procyanidin oligomer-rich extracts from different Cinnamon barks. Phytomedicine.

[22] Johny A.K., Darre M.J., Hoagland T.A., Schreiber D.T., Donoghue A.M., Donoghue D.J., Venkitanarayanan K. (2008). Antibacterial effect of trans-cinnamaldehyde on Salmonella enteritidis and Campylobacter jejuni in chicken drinking water. J. Appl. Poult. Res..

[23] Mishra A., Bhatti R., Singh A., Singh Ishar M.P. (2009). Ameliorative effect of the cinnamon oil from Cinnamomum zeylanicum upon early stage diabetic nephropathy. Planta Med..

[24] Nassar-Abbas S.M., Halkman K. (2004). Antimicrobial effect of water extract of sumac (*Rhus coriaria* L.) on the growth of some food borne bacteria including pathogens. Int. J. Food Microbiol..

[25] (2013). Performance Standards for Antimicrobial Susceptibility Testing.

[26] Ismaili H., Milella L., Fkih-Tetouani S., Ilidrissi A., Camporese A., Sosa S., Altinier G., Della Loggia R., Aquino R. (2004). In vivo topical anti-inflammatory and in vitro antioxidant activities of two extracts of Thymus satureioides leaves. J. Ethnopharmacol..

[27] Rukayadi Y., Han S., Yong D., Hwang J.K. (2010). In vitro antibacterial activity of panduratin A against enterococci clinical isolates. Biol. Pharm. Bull..

[28] MacFie H.J., Bratchell N., Greenhoff K., Vallis L.V. (1989). Designs to balance the effect of order of presentation and first-order carry-over effects in hall tests. J. Sens. Stud..

[29] (1988). Sensory Analysis—General Guidance for the Design of Test Rooms.

[30] Weerakkody N.S., Caffin N., Turner M.S., Dykes G.A. (2010). In vitro antimicrobial activity of less-utilized spice and herb extracts against selected food-borne bacteria. Food Control.

[31] Norhana M.W., Poole S.E., Deeth H.C., Dykes G.A. (2009). Effects of bilimbi (*Averrhoa bilimbi* L.) and tamarind (*Tamarindus indica* L.) juice on Listeria monocytogenes Scott A and Salmonella Typhimurium ATCC 14028 and the sensory properties of raw shrimps. Int. J. Food Microbiol..

[32] Pandey S., Pandey R., Singh R. (2014). Phytochemical screening of selected medicinal plant cinnamon zeylanicum bark extract, area of research; uttarakhand, India. Int. J. Sci. Res. Publ..

[33] Hacioglu M., Dosler S., Tan A.S.B., Otuk G. (2017). Antimicrobial activities of widely consumed herbal teas, alone or in combination with antibiotics: An in vitro study. PeerJ.

[34] Gilani S., Najafpour G. (2022). Evaluation of the extraction process parameters on bioactive compounds of cinnamon bark: A comparative study. Process Biochem..

[35] Sana S., Arshad M.U., Farhan S., Ahmad R., Ali I., Tabussam T. (2019). Nutritional characterization of cinnamon and turmeric with special reference to their antioxidant profile. Int. J. Biosci..

[36] Huang D., Ou B., Prior R.L. (2005). The chemistry behind antioxidant capacity assays. J. Agric. Food Chem..

[37] Madsen H.L., Bertelsen G. (1995). Spices as antioxidants. Trends Food Sci. Technol..

[38] Bajpai V.K., Sharma A., Baek K.H. (2013). Antibacterial mode of action of Cudrania tricuspidata fruit essential oil, affecting membrane permeability and surface characteristics of food-borne pathogens. Food Control.

[39] Gutierrez J., Barry-Ryan C., Bourke P. (2008). The antimicrobial efficacy of plant essential oil combinations and interactions with food ingredients. Int. J. Food Microbiol..

[40] González-Fandos E., Herrera B., Maya N. (2009). Efficacy of citric acid against Listeria monocytogenes attached to poultry skin during refrigerated storage. Int. J. Food Sci. Technol..

[41] Hassan M.A., Ibrahim H.M., Shawky N.A., Sheir S.H. (2020). Incidence of Psychotropic bacteria in frozen chicken meat products with special reference to *Pseudomonas* species. Benha Vet. Med. J..

[42] Elbehiry A., Marzouk E., Aldubaib M., Moussa I., Abalkhail A., Ibrahem M., Hamada M., Sindi W., Alzaben F., Almuzaini A.M. (2022). Pseudomonas species prevalence, protein analysis, and antibiotic resistance: An evolving public health challenge. AMB Express.

[43] Zhao Y., Wells J.H., McMillin K.W. (1994). Applications of dynamic modified atmosphere packaging systems for fresh red meats: Review. J. Muscle Foods.

[44] Jamilah M.B., Abbas K.A., Rahman R.A. (2008). A Review on Some Organic Acids Additives as Shelf Life Extenders of Fresh Beef Cuts. Am. J. Agric. Biol. Sci..

[45] Burt S. (2004). Essential oils: Their antibacterial properties and potential applications in foods—A review. Int. J. Food Microbiol..

[46] Bouhdid S., Abrini J., Amensour M., Zhiri A., Espuny M.J., Manresa A. (2010). Functional and ultrastructural changes in Pseudomonas aeruginosa and Staphylococcus aureus cells induced by *Cinnamomum verum* essential oil. J. Appl. Microbiol..

[47] Shange N., Makasi T., Gouws P., Hoffman L.C. (2019). Preservation of previously frozen black wildebeest meat (Connochaetes gnou) using oregano (*Oreganum vulgare*) essential oil. Meat Sci..

[48] Hussain Z., Li X., Zhang D., Hou C., Ijaz M., Bai Y., Xiao X., Zheng X. (2021). Influence of adding cinnamon bark oil on meat quality of ground lamb during storage at 4 C. Meat Sci..

[49] Van Haute S., Raes K., Van Der Meeren P., Sampers I. (2016). The effect of cinnamon, oregano and thyme essential oils in marinade on the microbial shelf life of fish and meat products. Food Control.

[50] Putra A.A., Wattanachant S., Wattanachant C. (2019). Sensory-related attributes of raw and cooked meat of culled Saanen goat marinated in ginger and pineapple juices. Trop. Anim. Sci. J..

[51] García-Casal M.N., Peña-Rosas J.P., Malavé H. (2016). Sauces, spices, and condiments: Definitions, potential benefits, consumption patterns, and global markets. Ann. N. Y. Acad. Sci..

